# Hamartoma of the soft palate: Case report and review of literature

**DOI:** 10.1016/j.ijscr.2024.109281

**Published:** 2024-01-20

**Authors:** El Krimi Zineb, Saoutarrih Badr, Douimi Loubna, Bijou Walid, Mahtar Mohamed

**Affiliations:** Department of Otolaryngology, Head and Neck Surgery, Ibn Rochd University Hospital, Faculty of Medicine and Pharmacy, Hassan II University, Casablanca, Morocco

**Keywords:** Hamartoma, Congenital deformity, Soft palate, Case report, Diagnosis

## Abstract

**Introduction:**

Hamartoma is a tumor-like malformation that represents a focal proliferation of normal cells. Hamartoma of the soft palate is a rare entity, which can lead to serious, life-threatening clinical manifestations, given its anatomical location. However, if properly treated surgically, their prognosis is excellent.

**Case presentation:**

The literature reports very few similar cases. So, we report a case of hamartoma of the soft palate, which presented as a sessile velar outgrowth arising on the midline of a 12-day-old newborn. The final diagnosis was based on histopathology. The patient was treated surgically and had excellent evolution.

Clinical Discussion.

Clinical examination shows hamartomas of the palate to be polyploid lesions, with a firm surface. A CT scan and magnetic resonance imaging (MRI) are indicated to establish the extent of the tumor. The diagnosis of certainty is determined by a histopathological examination. The only treatment is surgery, generally via the transoral approach. Prognosis is excellent.

**Conclusion:**

Hamartomas of the palate are diagnosed histologically, with imaging being of great help in assessing extension, and their therapeutic management is exclusively surgical.

The prognosis after successful surgery is practically always good, with no recurrence.

## Highlights

• Hamartoma is a tumor-like malformation, comprising a disorganized combination of mature tissues gradually increasing in volume.
• Hamartomas of the soft palate remain a rare entity, whose anatomical location may lead to clinically serious complications.
• The palate hamartoma is clinically described as a polyploid lesion.
• CT scan and magnetic resonance imaging are indicated to determine the extent of the lesion. The diagnosis of certainty is determined by histopathological examination.
• Surgery, usually transoral, is the only treatment available. Its prognosis is excellent.

## 1 Introduction

Hamartoma is derived from the Greek word hamartia, meaning defect. It's a non-neoplastic structural deformity, involving a cytologically normal mixture of mature cells and tissues that are autochthonous to the anatomical location, presenting a disorganized architectural pattern with predominance of one of its components [1].

In the oral cavity, such defects are usually located on the tongue near the cecum foramen, or on the anterior hard palate near the incisive papilla. Most are isolated phenomena, although a small number may be associated with other developmental defects or with a complex syndrome [[2], [3]].

.Clinically, hamartomas appear as hard, firm, pedicle-like masses that can lead to varying degrees of obstruction of the lumen opposite, without invading neighboring anatomical structures. Only histopathological examination allows a diagnosis of certainty. Treatment is purely surgical, with a very good prognosis [[1], [2]].

We report a case of hamartoma of the soft palate of a 12-day-old newborn managed in our department.

This case report has been reported in line with the SCARE criteria [13].

## 2 Case report

We report the case of a 12 days old newborn, who consulted our ENT department for a respiratory gene consisting mainly of nocturnal swelling and a gene during swallowing, noticed by the parents since the first day of the newborn's life.

Referring back to the archives. This is the only case of hamartoma of the palate seen in the neonatology department.

**Fig. 1:**
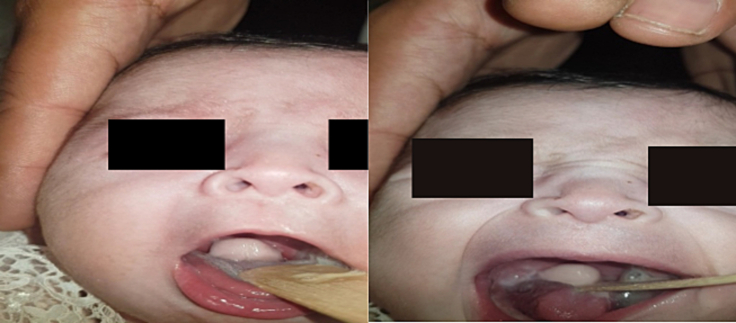
Clinical presentation of hamartoma of soft palate.

On clinical examination, the newborn was conscious with an oxygen saturation of 100 %, and signs of suprasternal tugging. Examination of the oral cavity revealed a white pediculated mass in the oropharynx (Fig. 1).

**Fig. 2:**
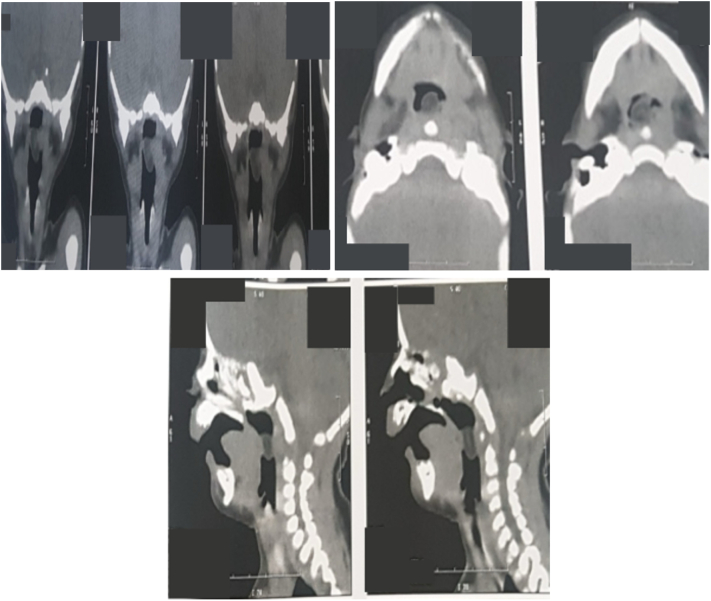
CT scan showing hamartoma of the soft palate as a mass of mixed tissue and fat density measuring 5.9 × 6.6 × 13.7 mm, filling the lumen in front of and respecting the valleculas, base of tongue and prevertebral space.

Once the patient was stabilized, a CT scan was performed, revealing a mass of mixed tissue and fat density in the oropharynx measuring 5.9 × 6.6 × 13.7 mm, filling the lumen in front of and respecting the valleculas, base of tongue and prevertebral space (Fig. 2).

The therapeutic decision was to perform a complete surgical resection of the mass via the transoral approach performed by a pediatric surgeon.

The main preoperative challenges reported by the surgeon were the bleeding, which he was able to control with electric cauterization, and the loss of tissue resulting from the removal of the lesion. The strategy adopted by the surgeon was to suture the nasal mucosa and buccal mucosa, layer by layer.

Pathological examination of the surgical specimen revealed a polypoid lesion with a thinned hyper-orthokeratosis epidermis opposite a dermis occupied by adipose lobules interspersed with fibrous trabeculae. This is consistent with a fibro-lipomatous hamartoma, with no signs of malignancy.

The main post-operative challenge was to avoid suture loosening, which is why we insisted on a cold and liquid diet for 15 days.

The evolution was excellent in particular no post-operative complications or recurrence, and the most important disappearance of the respiratory gene.

## 3 Discussion

Hamartomas were first described by Eugen Albrecht, a German pathologist, in 1904 [7].

Numerous medical research describes hamartoma as a congenital disorder. However, some hamartomas are only diagnosed at an advanced age. The disorder comprises a tumor-like deformity, comprising a disorganized combination of mature tissues appearing in the affected region and gradually increasing in volume [1,3].

The oral cavity hamartoma is rare, mainly dominated by hemangioma and lymphangioma. Their main location is the tongue [2]. Overall, most hamartomas affect men more often than women. Hamartomas affect any organ, but essentially the spleen, liver, kidney, lung and pancreas [3].

Some genes such as SMAD4, PTEN, STK1 and BMPR1A have been implicated in hamartoma development. Hamartoma-related disorders and conditions include tuberous sclerosis, Cowden's syndrome, PTEN hamartoma tumor syndrome and Peutz-Jeghers syndrome [5].

The development of the palate begins at the end of the 5th week of embryonic gestation and ends in the 12th week.

Clinical examination shows hamartomas of the palate as polyploid, sessile or pedicle-shaped lesions with a lobulated, spherical and firm surface. A detailed family history is important for determining associated diseases [6]. The early diagnosis of oral cavity hamartomas helps prevent complications, such as dyspnea or dysphagia.

Usually, hamartomas in other locations are asymptomatic. However, in some cases such as hypothalamic hamartomas and pulmonary hamartomas, the symptoms may be more marked. In the case of hypothalamic hamartomas, gelastic seizures, visual disturbances, early onset of puberty and behavioral disorders are the most frequently reported [8].

To assess the tumor's extension, CT and magnetic resonance imaging (MRI) are indicated. The radiological findings are variable, reflecting a solid tissue lesion that is fairly well delimited, with variable density depending on the lesioned constituents, and without bone lysis or invasion of surrounding organs [9]. Notably, MRI is the preferred imaging technique for the evaluation of hypothalamic, spleen, kidney and other abdominal hamartomas. On MRI, the hamartoma is characterized by a heterogeneous signal in T1 and a high signal due to fatty and cartilaginous components in T2 [8]. Histologically, it is characterized by a hyperplastic mixture, in variable proportions, of different benign tissue elements, such as fibrous tissue, smooth or striated muscle, adipose tissue, lymphoid tissue, nerve tissue and bone; there are also salivary glands and blood vessels [10].

The clinical differential diagnosis can be made with essentially a teratoma, a congenital epulis, a leiomyoma or a tumor of the accessory salivary glands [11].

Congenital tumors of the oral cavity can affect the normal development of adjacent structures such as palate. In fact, congenital tumors developing during morphogenesis of the palate may be associated with cleft palate, lobulated tongue or micro-phthalmia [4].

Hamartomas are not medically treatable; the main therapy is surgical treatment. If the lesion is asymptomatic and of small volume, surgical intervention is not necessary, unless there is a medical emergency involving dyspnea or dysphagia [12].

In general, the prognosis is favorable, recurrences are rare, and are mostly due to incomplete excision [12].

## 4 Conclusion

Hamartomas of the oral cavity are benign malformations, represented essentially by vascular malformations such as hemangiomas and lymphangiomas. However, hamartomas formed by other types of tissue are much less common in the oral cavity, and are reported only very rarely in the palate. Clinically it may mimic malignant neoplasia. The diagnosis is histological and Its treatment is essentially surgical.

The prognosis is always favorable, with no recurrence.

## Consent

Written informed consent was obtained from the patient's parents/legal guardian for publication of this case report and accompanying images. A copy of the written consent is available for review by the Editor-in-Chief of this journal on request.

## Ethical approval

Ethics approval is not required for case reports deemed not to constitute research in our institution.

## Funding

None.

## Author contribution

Elkrimi Zineb: Study concept and writing the paper.

Saoutarrih Badr: Corresponding author and writing the paper.

Douimi Loubna: Study concept and writing the paper.

Bijou Walid: Study concept and correction of the paper.

Mahtar Mohamed: Study concept and correction of the paper.

## Guarantor

Elkrimi Zineb.

## Research registration number

N/a.

## Provenance and peer review

Not commissioned, externally peer-reviewed.

## Conflict of interest statement

The authors declare having no conflicts of interest for this article.
